# Tailoring Midazolam-Loaded Chitosan Nanoparticulate Formulation for Enhanced Brain Delivery via Intranasal Route

**DOI:** 10.3390/polym12112589

**Published:** 2020-11-04

**Authors:** Nikesh Shrestha, Saba Khan, Yub Raj Neupane, Shweta Dang, Shadab Md, Usama A. Fahmy, Sabna Kotta, Nabil A. Alhakamy, Sanjula Baboota, Javed Ali

**Affiliations:** 1 Department of Pharmaceutics, School of Pharmaceutical Education and Research, Jamia Hamdard, New Delhi 110062, India; phar.nikeshstha@gmail.com (N.S.); sabakhan027@gmail.com (S.K.); sbaboota@jamiahamdard.ac.in (S.B.); 2 Department of Pharmacy, National University of Singapore, Singapore 117559, Singapore; yubraj9841@gmail.com; 3 Department of Biotechnology, Jaypee Institute of Information Technology, Noida 201309, Uttar Pradesh, India; shweta.dang@jiit.ac.in; 4 Department of Pharmaceutics, Faculty of Pharmacy, King Abdulaziz University, Jeddah 21589, Saudi Arabia; shaque@kau.edu.sa (S.M.); uahmedkauedu.sa@kau.edu.sa (U.A.F.); skotta@kau.edu.sa (S.K.); nalhakamy@kau.edu.sa (N.A.A.); 5 Center of Excellence for Drug Research & Pharmaceutical Industries, King Abdulaziz University, Jeddah 21589, Saudi Arabia

**Keywords:** midazolam, status epilepticus, chitosan, nanoparticles, ionic gelation method, intranasal route, brain delivery

## Abstract

In the present study, midazolam (MDZ)-loaded chitosan nanoparticle formulation was investigated for enhanced transport to the brain through the intranasal (IN) route. These days, IN MDZ is very much in demand for treating life-threatening seizure emergencies; therefore, its nanoparticle formulation was formulated in the present work because it could substantially improve its brain targeting via the IN route. MDZ-loaded chitosan nanoparticles (MDZ-CSNPs) were formulated and optimized by the ionic gelation method and then evaluated for particle size, particle size distribution (PDI), drug loading (DL), encapsulation efficiency (EE), and in vitro release as well as in vitro permeation. The concentration of MDZ in the brain after the intranasal administration of MDZ-CSNPs (*C*_max_ 423.41 ± 10.23 ng/mL, tmax 2 h, and area under the curve from 0 to 480 min (AUC_0-480)_ of 1920.87 ng.min/mL) was found to be comparatively higher to that achieved following intravenous (IV) administration of MDZ solution (*C*_max_ 245.44 ± 12.83 ng/mL, t_max_ 1 h, and AUC_0-480_ 1208.94 ng.min/mL) and IN administration of MDZ solution (*C*_max_ 211.67 ± 12.82, t_max_ 2 h, and AUC_0-480_ 1036.78 ng.min/mL). The brain–blood ratio of MDZ-CSNPs (IN) were significantly greater at all sampling time points when compared to that of MDZ solution (IV) and MDZ (IN), which indicate that direct nose-to-brain delivery by bypassing the blood–brain barrier demonstrates superiority in brain delivery. The drug-targeting efficiency (DTE%) as well as nose-to-brain direct transport percentage (DTP%) of MDZ-CSNPs (IN) was found to be comparatively higher than that for other formulations, suggesting better brain targeting potential. Thus, the obtained results demonstrated that IN MDZ-CSNP has come up as a promising approach, which exhibits tremendous potential to mark a new landscape for the treatment of status epilepticus.

## 1. Introduction

Status Epilepticus is a serious and life-threatening condition with a substantially high mortality and morbidity rate in survivors if not expeditiously sacked [1]. For treating these dreadful seizures episodes, the anti-epileptics such as benzodiazepines are extensively needed to be rapidly delivered to the brain. In such circumstances, the nasal route has come up as the savior attributed to its speedy and uninterrupted direct drug transport to the brain. The major advantages of this route are its rich vascularity, non-invasiveness, absence of intestinal/liver metabolism, and relatively greater absorptive surface area. Nevertheless, certain limitations are there, such as the restricted capacity of the nasal cavity and poor water solubility of the anti-epileptic drugs that confine the absorption, resulting in suboptimal therapeutic brain levels. Therefore, the formulation strategy is needed to be employed to address these obstacles and enhance the nasal delivery of therapeutics, particularly benzodiazepines, which are used to treat seizure emergency conditions.

Intranasal midazolam (MDZ) is an emerging potential technique for periprocedural sedation and for acute seizure control. The most lucrative feature it offers is that such intranasal (IN) formulation allows for rapid and easy administration. MDZ’s potency and efficacy make it an excellent drug for treating Status Epilepticus. MDZ (chemically 8-chloro-6-(-2-fluorophenyl)-1-methyl-4H-imidzao[1,5,-a][1,4] benzodiazepine) is a water-soluble benzodiazepine [2], which exerts its anti-epileptic action by binding to the receptor gamma-aminobutyric acid (GABA) at its binding site positioned between the α subunit and γ subunit, which results in increased affinity for GABA, thereby increasing the frequency of the chloride channel opening [1,3]. It is among the first line drugs for the treatment of Status Epilepticus (SE). However, the high water solubility, high rate of hepatic first pass metabolism, and short biological half-life [4] limit its clinical application in the treatment of SE [5]. In the present study, MDZ was selected as the drug candidate whose polymeric nanoparticle formulation was investigated for improved delivery to the brain through the nasal route.

In the current scenario, MDZ is available in the market only as an injectable and oral syrup; nonetheless, it is the most extensively researched intranasal benzodiazepine. In the preceding studies, in humans, the calculated bioavailability of MDZ after intranasal administration has ranged from 50 to 83% [6,7]. In most of these studies, a dilute aqueous solution of MDZ injection was employed that was neither appropriate for intranasal administration due to low pH nor was it able to gain optimal therapeutic brain concentration due to nasal run-off. The major reason for such failures were that those formulations are too diluted for nasal administration; thus, the required dose of MDZ that was diluted within that volume and was not able to get delivered to the brain [6,7]. Therefore, a formulation strategy is highly needed to address this unmet medical need for the development of an optimal MDZ formulation for intranasal delivery. In light of these studies, in the present study, chitosan (CS) is selected as a polymer for the fabrication of polymeric nanoparticles (PNPs) to overcome the limitation of the intranasal route. The PNPs approach is an approach that has been used for the delivery of therapeutics into the brain since nanoparticles can facilitate the transport of drugs across blood–brain barrier (BBB). This strategy can protect the encapsulated therapeutic agent from degradation and efflux by P-gp efflux transporters [8]. Transcellular transport by olfactory neurons to the brain by different endocytic pathways by olfactory membrane sustentacular or neuronal cells can be well achieved by these nanoparticles due to their small diameter [9].

Furthermore, the low permeability of nasal mucosa and nasal mucociliary clearance are the two restrictive factors for the nasal delivery of therapeutics, which hinders drug absorption [10]. Mucocillary clearance can be decreased by the use of mucoadhesive polymer such as CS [11], which in turn would increase the residence time in nasal mucosa and thereby allow a longer time for absorption and help gain more intimate contact with the nasal mucosa, which would result in a subsequent increase in drug absorption and transport to the brain [12,13].

Chitosan has been widely researched in brain drug delivery after the development of chitosan microparticles by Gallo in 1993 [14]. Chitosan is the only natural alkaline polysaccharide that possesses excellent biocompatibility as well as biodegradability. Nanoparticles made from chitosan for encapsulating therapeutic moieties have been proved with advantages of protection from enzyme degradation, controlled release, as well as superior bioavailability. Moreover, it can improve drug permeability across the BBB by affecting the tight junction. The residence time on the nasal mucosa is high because of the interaction of positive charge on the surface with a negatively charged cell membrane as a result of the improved delivery of drugs from the nasal cavity to the brain [15]. Chitosan is extensively researched for nose to brain targeting of therapeutics as in the form of nanoparticles. Bari et al. formulated and evaluated thiolated chitosan nanoparticles of buspirone hydrochloride for brain delivery by the intranasal route. The formulation showed a drug entrapment efficiency of 81.13 and a loading capacity of 49.67% [16]. Tzeyung et al. developed and evaluated rotigotine-loaded chitosan nanoparticles for nose-to-brain delivery [17]. Bhattamisra and associates also investigated nose-to-brain delivery of rotigotine-loaded chitosan nanoparticles in an in vitro cell line and in an animal model of Parkinson’s disease. The optimized formulation showed improved efficacy and brain targeting through the intranasal route [18]. Meng and co-workers developed Huperzine A-loaded mucoadhesive and targeted N-trimethylated chitosan-conjugated poly(lactic-co-glycolic acid) (PLGA) (PLGA) nanoparticles for the treatment of Alzheimer’s disease through the intranasal route [19]. Sridhar and associates developed selegiline-loaded chitosan nanoparticles with more than 90% drug loading and steady in vitro and ex vivo drug release, which showed 20 and 12-fold drug concentrations in the brain and plasma respectively, after intranasal administration as compared to oral administration [20]. In a recent work, Jahromi and associates developed methotrexate-loaded chitosan-based hydrogel nanoparticles for central nervous system (CNS) drug delivery. The optimized formulation showed a drug-loading efficiency of 72.03 ± 0.85 and produced a significantly higher brain concentration compared with the simple solution [21]. Raj and co-workers developed pramipexole dihydrochloride-loaded chitosan nanoparticles for Parkinson disease via the nose-to-brain pathway. The optimized formulation showed satisfactory particle size, higher entrapment efficiency 91 ± 0.95%, and exhibited superior in vivo activity as compared to the conventional formulation [22].

Chitosan is approved by the United States-Food and Drug Administration (US-FDA) and the European Union (EU) as safe for dietary use and wound-dressing applications. There have been no published data available on the human toxicity of formulations made of chitosan, and also, the clinical use of oral or mucoadhesive formulations from chitosan remain on the horizon [23,24]. However, there are already human vaccines with chitosan as an adjuvant under development. A chitosan–glutamate intranasal system as a diphtheria toxoid antigen was evaluated in healthy human volunteers for the safety and immunostimulatory effects and was found to be well tolerated [25]. Clinical trials on an intranasal vaccine containing chitosan as a spray-dried powder did not report any adverse effects, either [26].

Chitosan is the most studied polymer for accessing polymeric nanomaterial toxicity. There are several research studies published regarding the in vivo toxicity of chitosan nanoparticles by various routes of administration. Studies were done to evaluate the toxicity of the chitosan-derived nanomaterials and to identify possible risks to human health; researchers performed in vivo tests in animals to evaluate acute and repeated-dose (subacute, sub-chronic, or chronic) toxicity. Satisfactory results were reported in many of the cases and were included in the review published on the Safe-by-Design concept by Jesus et al. [27]. In another review by Maques et al. regarding the implementation of the Safe-by-Design concept for evaluation of the efficacy and safety of nanomedicines points out the different characterization of chitosan done so far. From the research published so far regarding the toxicity of chitosan nanomaterials, it has been found that this polymer is promising for drug delivery applications [28].

The objectives of the present work were to formulate and characterize MDZ-loaded chitosan nanoparticles (MDZ-CSNPs) and also to evaluate on the basis of pharmacokinetic study parameters. The MDZ concentration in brain after the intranasal administration of MDZ-CSNP was compared with IV and IN administration of MDZ solution. The brain–blood ratio of MDZ-CSNP (IN) was compared to MDZ solution (IV) and MDZ (IN) indicated that direct nose-to-brain delivery by bypassing the BBB for achieving superiority in brain delivery. The drug-targeting efficiency (DTE%) as well direct transport percentage (DTP%) from nose to brain of MDZ-CSNP (IN) were compared and contrasted with the other formulations to evaluate the efficiency in brain targeting.

## 2. Materials and Methods

### 2.1. Materials

MDZ was procured from R.L. Fine Chem, Bangalore, India. Chitosan and sodium tripolyphosphate were procured from Sigma-Aldrich, Bangalore, India. Glacial acetic acid was obtained from IOL Chemical Ltd., Mumbai, India. HPLC grade methanol, acetonitrile, and water were procured from S.D. Chemicals, Mumbai, India. Dialysis sacs (mol. wt. cut-off: 12,000 Da) were procured from Sigma–Aldrich, St. Louis, MO, USA. All other reagents and chemicals used in this experiment were of analytical reagent grade.

### 2.2. Methods

#### 2.2.1. Preparation of Chitosan Nanoparticles

The chitosan nanoparticles were formulated by the ionic gelation of chitosan and sodium tripolyphosphate (Na-TPP) as a cross-linking agent [29,30]. Briefly, chitosan was dissolved in glacial acetic acid (2% *v/v*) in different concentrations of chitosan (0.05, 0.075, 0.1, 0.15, 0.175, 0.2 and 0.25% *w/v*). Different concentrations of (0.1, 0.15, 0.2, 0.3% *w/v*) Na-TPP were dissolved in distilled water. From these solutions, 4 mL of Na-TPP solution was drop-wise mixed to 10 mL of chitosan solution at room temperature with constant stirring. NPs formation was observed only for some specific concentration of CS and Na-TPP. After addition, the mixture was kept on a magnetic stirrer with continuous stirring for 30 min. The drug was loaded into NPs by adding it to the chitosan solution in different ratios (1:1, 1:2, and 1:3) and kept aside undisturbed for 24 h before adding Na-TPP solution. Ultracentrifugation (at 20,000× *g* at 10 °C for 45 min) was done to separate the resultant NPs from the aqueous medium. Entrapment efficiency and drug loading were determined by analyzing the supernatant drug content. The resultant NPs were freeze-dried after washing with distilled water.

#### 2.2.2. Physiochemical Characterization of Chitosan Nanoparticles

The size and size distribution of the nanoparticles were measured by Zetasizer Nano ZS, (Malvern Instruments Ltd., Worcestershire, UK). One mL of sample was used for the analysis. Since all the particles in the medium exhibit Brownian motion, scattering of light will occur, and the changes in intensity of light will detect suitable optics and a photo multiplier.

Transmission electron microscopy (TEM) was used for further evaluation of the particle size and surface morphology for the optimized nanoparticles (Morgagni 268D TEM, FEI, Hillsboro, OR, USA) at an acceleration voltage of 200 kV and viewed at a magnification of 50,000× *g*. A drop of the diluted sample of the nanoparticle was placed on a copper grid and stained with phosphotungstic acid, and after complete drying, the sample was analyzed, and images were captured [31].

#### 2.2.3. Entrapment Efficiency (EE) and Loading Capacity (LC) of MDZ-Loaded Chitosan Nanoparticles

To determine the EE and LC of prepared nanoparticles, the formulations were centrifuged at 20,000× *g* at 10 °C for 45 min using Beckman Coulter Optima LE-80K Ultracentrifuge, and the supernatant was filtered (0.45 μm filter) and drug concentration was calculated by UV spectrophotometer. The below given equations were used to calculate the EE and LC of MDZ-loaded CS NPs. The average of three measurements was taken.

(1) $$\mathrm{EE}=\frac{\mathrm{Total}\;\mathrm{Drug}\;\mathrm{loaded}-\mathrm{Free}\;\mathrm{Drug}\;\mathrm{in}\;\mathrm{solution}}{\mathrm{Total}\;\mathrm{Drug}}\times 100$$

(2) $$\mathrm{LC}=\frac{\mathrm{Total}\;\mathrm{Drug}\;\mathrm{loaded}-\mathrm{Free}\;\mathrm{Drug}\;\mathrm{in}\;\mathrm{solution}}{\mathrm{Nanoparticles}\;\mathrm{weight}}\times 100$$

#### 2.2.4. Differential Scanning Calorimetry (DSC)

The differential scanning calorimetry (DSC) analysis of pure MDZ and MDZ-loaded CSNPs was carried out using a Perkin Elmer 7 DSC (Boston, MA, USA) calibrated with indium. Five mg of sample was placed on aluminium pan, crimped, and heated from 40 to 400 °C with a continuous purging of nitrogen (20 mL/min). An empty sealed pan was used as reference.

### 2.3. In Vitro Release Study

Using dialysis membrane in vitro release profile of MDZ from MDZ-loaded CS NPs was carried out. To a pretreated dialysis bag, a 5 mL suspension of MDZ-loaded CS NPs (containing drug equivalent to 2 mg) was placed, which was then dipped into pH 7.4 phosphate buffer solution in a 100 mL beaker and magnetically stirred at 100 rpm. At predetermined time intervals, 5 mL of solution was taken out for analysis and was replaced by fresh dissolution media. Using a UV spectrophotometer at 221 nm, the concentration of released drug was determined, and the cumulative percentage drug release was calculated. In each experiment, the analysis was done in triplicate.

The data were fitted to different kinetic models such as zero-order, first-order, Higuchi model, and Korsmeyer–Peppas model to interpret the mechanism and kinetics of drug release [32].

### 2.4. In Vitro Permeation Studies

Fresh slices of nasal tissues were removed carefully from the nasal cavity of goat purchased from the nearby slaughterhouse. The nasal tissue (permeation area of 0.785 cm^2^) was placed properly in a Franz diffusion cell. The receptor chamber was filled with pH 6.4 phosphate buffer saline (PBS) (10 mL), and the temperature was maintained at 37 °C [16,33]. A mixture of O_2_ (95%) and CO_2_ (5%) was bubbled to ensure oxygenation and agitation in the system. Twenty min was allowed for equibriation of the system, and then 5 mL of drug solution and the formulation (equivalent to 0.5 mg of MDZ) was kept in the donor compartment. One mL of sample was withdrawn at each time point from the receptor chamber. The volume was replaced by fresh pH 6.4 PBS after each sampling time. The samples were taken until 12 h. The filtered samples were analyzed for permeated MDZ content at 221 nm using UV spectrophotometer.

### 2.5. In Vivo Studies

Albino Wistar rats of either sex weighing 200–250 g were selected for the biodistribution and pharmacokinetic studies. The study was conducted strictly under the guidelines for animal handling after getting approval from the Institutional Animal Ethics Committee of Jamia Hamdard (Protocol no: 1174). The animals were kept at 12 h light–dark cycle at a temperature of 20–24 °C with free access to food and water until the day of the experiment. Animals were divided into 3 groups, group A received IV MDZ solution, Group B received IN MDZ solution, and group C received IN MDZ-loaded CSNPs. For each time point (points of sampling (*n* = 3): 0.5, 1, 2, 4, and 8), three rats were used from each group.

Mild anesthesia was induced using diethyl ether before administering the formulations. Freeze-dried MDZ NPs suspended in saline buffer and drug solution (1.5 mg/kg body weight) were administered to rats of group B and C respectively via an IN route (20 μL in each nostril). The formulation was instilled using a micropipette. Intravenous MDZ solution was given to group A. Blood was collected from the retro-orbital vein at each time point in to ethylenediaminetetraacetic acid (EDTA)-coated tubes. After collecting the blood, rats were sacrificed by the cervical dislocation method. Afterwards, the brain was isolated and washed with normal saline thoroughly and made free from adhering tissue or fluid and then weighed. It was homogenized using 1% KCl, and drug was extracted using acetonitrile. This homogenate was centrifuged (5000 rpm for 15 min), the organic layer was carefully separated, and the drug content was analyzed by a pre-validated HPLC method (Shimadzu HPLC LC-10A VP equipped with 20 μL injector). The mobile phase was 10 mM phosphate buffer (pH 6.0) and acetonitrile in the ratio of 80:20 *v/v* using a column Li Chrospher C18 (250 mm × 4.6 mm internal diameter (i.d.), 5-μm particle) with a flow rate of 1 mL/min for a run time of 14 min.

Different pharmacokinetic parameters such as area under the curve (AUC), *T*_1/2_, *T*_max_, *K*_el_, and *C*_max_ were calculated using software PK Functions for Microsoft Excel (Pharsight Corporation, Mountain View, CA, USA). The *C*_max_ and *T*_max_ were directly noted from the actual plasma profiles. Using the below given equations, drug targeting efficiency (DTE%) that represents the time-average partitioning ratio and nose-to-brain direct transport percentage (DTP%) was calculated [34].

$$\mathrm{DTE}\%=\left[\frac{\left({\mathrm{AUC}}_{\mathrm{brain}}/{\mathrm{AUC}}_{\mathrm{blood}}\right)\mathrm{IN}}{\left({\mathrm{AUC}}_{\mathrm{brain}}/{\mathrm{AUC}}_{\mathrm{blood}}\right)\mathrm{IV}}\right]\times 100$$

$$\mathrm{DTP}\%=\left[\frac{B_{\mathrm{IN}}-B_{x}}{B_{\mathrm{IN}}}\right]\times 100$$

where Bx = (B_IV_/P_IV_) × P_IN_, (Bx is the brain AUC fraction contributed by systemic circulation through the BBB following IN administration); B_IN_ is the AUC_0–480_ (brain) following intravenous administration.; P_IV_ is the AUC_0–480_ (blood) following intravenous administration; B_IN_ is the AUC_0–480_ (brain) following IN administration; and P_IN_ is the AUC_0–480_ (blood) following IN administration

## 3. Results and Discussion

### 3.1. Formulation and Characterization of Chitosan Nanoparticles (CSNPs)

Different chitosan nanoparticles were formulated using different concentrations of chitosan and Na-TPP. Formulations showing opalescent behavior were selected and then observed for particle size and polydispersity index (PDI). The results are shown in Table 1.

**Figure 1:**
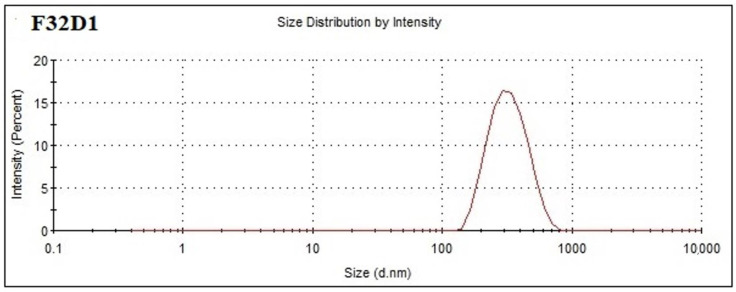
Particle size and polydispersity index (PDI) of MDZ-loaded chitosan nanoparticles (CS NPs) containing 1 mg/mL CS and 1 mg/mL sodium tripolyphosphate (Na-TPP) with drug:polymer ratio 1:1 (formulation F32 D1).

Out of all the formulations, the formulation F32 was selected for the preparation of MDZ-loaded CSNPs due to its optimum mean particle size (147.2 ± 2.21 nm) and PDI (0.268 ± 0.009), since a particle size less than 200 nm and PDI less than 0.5 is desirable for brain targeting through the nasal route. The other formulations were rejected due to their very large particles sizes and PDI, which makes them unsuitable for brain targeting via the intranasal route [17] Out of all formulations, the formulation F32 was selected for the preparation of MDZ-loaded CSNPs due to its optimum mean particle size (147.2 ± 2.21 nm) and PDI (0.268 ± 0.009). The mean particle size and PDI of MDZ-loaded chitosan nanoparticles containing 1 mg/mL of CS and 1 mg/mL of Na-TPP was determined using Zetasizer Nano ZS and found to be 146.7 ± 1.63 nm and 0.265 ± 0.005, respectively (Figure 1).

MDZ was incorporated into optimized chitosan nanoparticles F32 in the different drug-to-polymer ratio and MDZ-loaded CS NPs were examined for mean particle size, PDI, drug loading, and entrapment efficiency. The obtained results are shown in Table 2.

It was observed that as the drug/polymer ratio increases from 1:1 to 3:1, the mean particle size of MDZ-loaded CS NPs also increases from 241.20 ± 12.25 nm to 380.96 ±13.81 nm, as shown in Table 3, which indicated that there is a positive relationship between drug/polymer ratio and particle size owing to a decrease in interaction between CS and Na-TPP solution [35]. Out of three formulations, the optimum formulation F32 D1 was selected for further studies because of its optimum mean particle size (241.20 ± 12.25 nm), PDI (0.389 ± 0.056), EE% (88.68 ± 1.22), and DL% (36.45 ± 2.14). On increasing the drug to polymer ratio from 1:1 to 1:3, the entrapment efficiency decreased from 88.68 ± 1.22% to 68.46 ± 0.43 %. The excess amount of drug might have decreased the interaction between CS and Na-TPP, which thus in turn decreases the entrapment efficiency [36]. The result showed that a drug-to-polymer ratio of 1:1 has superior entrapment efficiency. The drug loading was observed to decrease from 36.45 ± 2.14 to 16.18 ± 2.01% with the increase in polymer ratio, as shown in Table 2.

**Figure 2:**
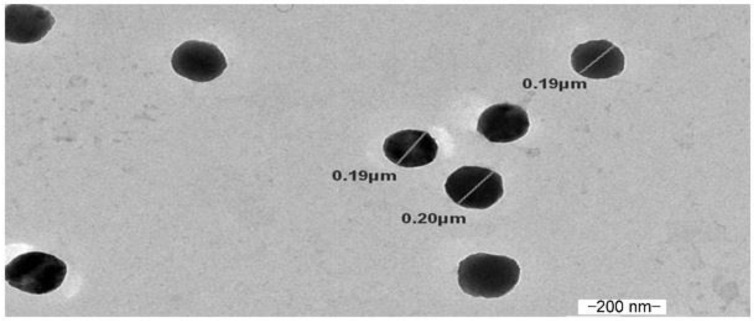
Transmission electron microscopy (TEM) image of MDZ-loaded CS NPs containing 1 mg/mL CS and 1 mg/mL Na-TPP (Formulation F32 D1).

The TEM image of optimized MDZ-loaded CS NPs is shown in Figure 2. The TEM image indicated that MDZ-loaded CS NPs formed by the ionic gelation method were spherical in shape, and the particle size ranged from 190 to 200 nm.

### 3.2. DSC

**Figure 3:**
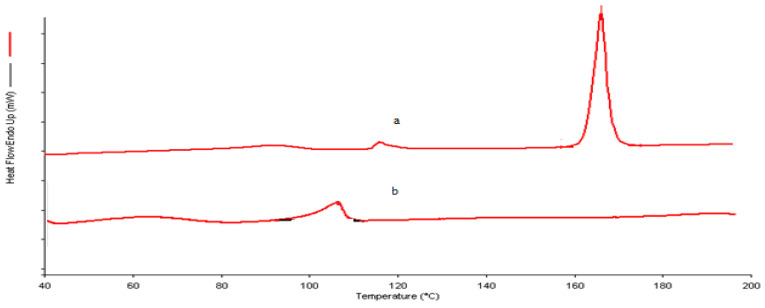
Differential scanning calorimetry (DSC) images of (**a**) MDZ and (**b**) MDZ-loaded CS NPs.

The DSC thermogram of MDZ showed an endothermic peak at 166.030 °C, as shown in Figure 3 with an enthalpy of 341.649 J/g, which showed that the sample provided is crystalline, whereas the DSC thermogram of MDZ-loaded CS NPs showed no endothermic peak of MDZ, which suggests that MDZ might have become dispersed within CS NPs or there was a loss of degree of crystallinity of the MDZ [37].

### 3.3. In Vitro Drug Release Study

**Figure 4:**
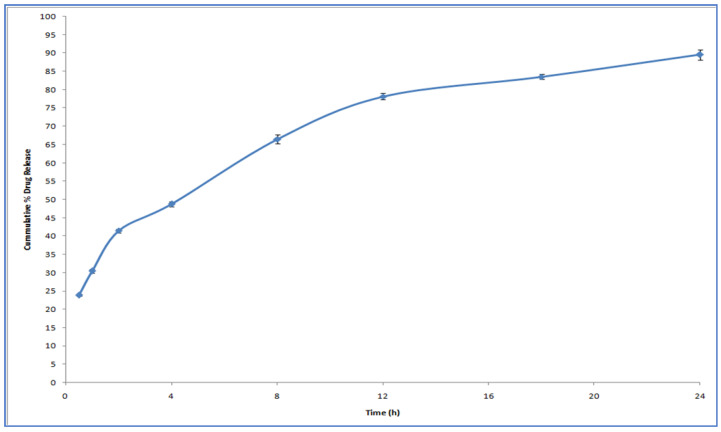
In vitro release profile of CS NPs loaded with MDZ (F32 D1).

Figure 4 describes the release profile of MDZ from CS NPs. We observed a rapid drug release for 1 h followed by slow release for 24 h. The initial rapid release of drug may be because of the drug adsorbed onto the NP surface, which dissolved when NPs came into contact with the release medium [38].

Due to the hydration and swelling of CS, the drug might be constantly released from the core of NPs through diffusion and erosion in later stages [39,40].

The data obtained from the in vitro release profile were fitted into standard release equations (zero, first, Higuchi, and Korsemeyer–Peppas model) to study the release kinetics. By analyzing the correlation coefficient value of the various models, the model that best fits the release data was selected. It was found that the release of the drug from NPs followed first-order release kinetics as indicated by a greater r^2^ value of 0.967. As indicated from the release data fitted in the Korsemeyer–Peppas equation, it was found that the drug release from CS NPs was by a non-Fickian diffusion mechanism, since the value of release exponent (*n*) was 0.589.

### 3.4. In Vitro Permeation Studies

The cumulative amount of the drug permeated (CAPD) through nasal mucosa from MDZ-loaded CS-NPs was 79.81%, whereas only 36% was found to permeate from pure drug solution. The steady-state flux and permeability coefficient of MDZ-loaded CS-NPs was 7.352 µg/cm^2^/h and 0.0147 cm^−2^h^−1^, respectively. However, for drug solution, the steady-state flux and permeability coefficient was only 3.424 µg/cm^2^/h and 0.0068 cm^−2^h^−1^, respectively. The CS NPs showed more permeability when compared with drug solution due to the presence of positively charged CS. The permeation enhancement by CS might be due to the transient opening of the epithelial cells in the nasal mucosa and also by attaching to the negative charges of sialic acid, which would consequently enhance the permeation of drug through nasal mucosa [41].

### 3.5. Study of Pharmacokinetic Parameters and Determination of Drug-Targeting Efficiency (DTE%) and Nose-to-Brain Direct Transport Percentage (DTP%)

PK Functions for Microsoft Excel (Pharsight Corporation, Mountain view, CA, USA) was used to calculate various pharmacokinetic parameters from plasma concentration–time profiles after IV and IN administration. Various pharmacokinetic parameters of MDZ formulation after IN and IV administration in rats are shown in Table 3.

The biodistribution of MDZ in brain and blood following IN administration of MDZ CS NPs, IN administration of MDZ solution, and IV administration of MDZ solution is shown in Table 4. Concentration of the drug at different time intervals up to 8 h was estimated using HPLC.

**Figure 5:**
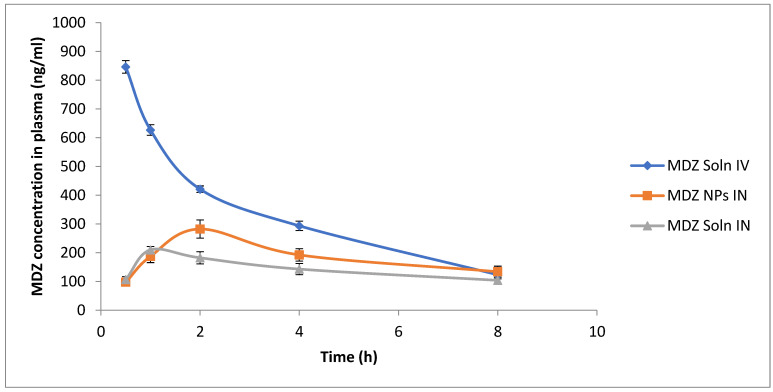
Graph of MDZ concentration in plasma at different time intervals following MDZ solution intravenous (IV), MDZ solution intranasal (IN), and MDZ CS NPs IN administration (*n* = 3, mean ± S.D.).

**Figure 6:**
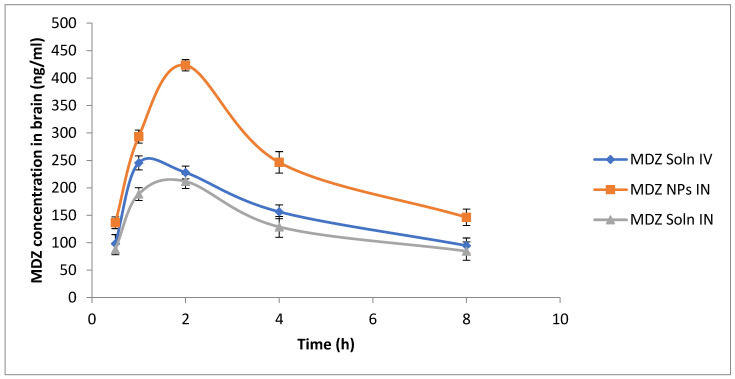
Graph of MDZ concentration in brain homogenate at different time intervals following MDZ solution IV, MDZ solution IN, and MDZ CS NPs IN administration (*n* = 3, mean ± S.D.).

Blood and brain concentration of MDZ vs. time following IV or IN administration of different formulations are shown in Figure 5 and Figure 6. When MDZ solution was administered IV, the *C*_max_ (846.32 ± 22.01 ng/mL) was reached at 0.5 h in blood followed by an exponential decrease in concentration, whereas following the IN administration of MDZ, CS NPs showed slow absorption through the nasal mucosal membrane into systemic circulation and *C*_max_ (282.34 ± 31.45 ng/mL) was reached at 2 h in blood, which is comparatively lower than *C*_max_ achieved after IV administration of MDZ solution (Figure 6). When MDZ solution was administered IV in the brain, the *C*_max_ (245.44 ± 12.83 ng/mL) was achieved at 1 h and the AUC_0-480_ was 1208.94 ng.min/mL. Comparatively, IN-administered MDZ CS NPs showed better absorption across the nasal mucosa into olfactory epithelium and *C*_max_ (423.41 ± 10.23 ng/mL) within 2 h, and AUC_0-480_ of 1920.87 ng.min/mL was achieved, which is comparatively higher compared to that achieved following an IV administration of MDZ solution. The brain–blood ratio was determined at sampling points for different formulations and is shown in Table 5.

The brain–blood ratio of MDZ-loaded CS NPs (IN) was significantly higher at all time points when compared to that of MDZ solution (IV) and MDZ solution (IN), which indicates that direct nose-to-brain transport through bypassing BBB and superiority in brain delivery following IN administration of MDZ-loaded CS NPs. These findings are similar to the results obtained for Zhang et al. [42].

The DTE% and DTP% represented the amount of drug directly conveyed to the brain through the olfactory pathway. After IV and IN administration, DTP% and DTE% were calculated using tissue/organ distribution data. The DTE% and DTP% of MDZ-loaded CSNPs through the IN route were found to be comparatively greater than that for MDZ IN solution, as shown in Table 6, which suggested better brain targeting after the administration of MDZ-loaded CS NPs through the intranasal route.

Hence, the overall result showed that MDZ concentration in the brain following IN administration of MDZ CS NPs was significantly higher compared to both IV and IN administration of MDZ solution. In addition, the brain–blood ratio of MDZ concentration at all the time points was higher for the IN administration of MDZ CS NPs compared to both IV and IN administrations of MDZ solution. At all the time points, better brain targeting was achieved after IN administration of MDZ-loaded CS NPs. This may be due to (1) the mucoadhesive nature of CS, which prolongs the nasal residence time in the nasal cavity by decreasing mucocilliary clearance [43]; (2) the permeation-enhancing activity of chitosan, which will transiently open the tight junctions between cells, which in turn will enhance paracellular transport [13]; and also (3) advantages of NPs such as protection against biological and or chemical degradation and the small diameter of NPs, which enhances transcellular transport through olfactory neurons to the brains [9].

## 4. Conclusions

A substantially higher brain concentration of MDZ when MDZ-loaded CS NPs were administered through IN route, and its greater DTE% and DTP% as compared to MDZ solution, clearly demonstrated that CSNPs have better MDZ brain-targeting efficiency through the IN route of administration. In our study, the nose-to-brain delivery of MDZ-loaded CSNPs was established with superiority over other formulations and other routes of administration. The resulting NPs effectively increased the brain uptake of MDZ via MDZ-loaded CSNPs. Thus, an effective non-invasive brain drug delivery system of MDZ for the management of SE has paved a way at therapeutic front; however, further clinical studies of the prepared formulation are needed to establish the clinical efficacy and evaluate the risk-to-benefit ratio.

## Figures and Tables

**Table 1:** Optimization of chitosan nanoparticles.

Formulation Code	CS (mg/mL)	Na-TPP (mg/mL)	Particle Size (nm) ± SD	PDI ± SD
F32	1	1	147.2 ± 2.21	0.268 ± 0.009
F41	1.5	1	188.4 ± 1.09	0.391 ± 0.028
F42	1.5	1.5	289.80 ± 3.87	0.288 ± 0.016
F51	1.75	1	205.0 ± 5.76	0.419 ± 0.007
F52	1.75	1.5	238.5 ± 2.86	0.550 ± 0.012
F61	2	1	440.6 ± 4.93	0.419 ± 0.017
F62	2	1.5	280.60 ± 2.1	0.389 ± 0.006
F71	2.5	1.5	325.6 ± 1.82	0.347 ± 0.013

CS: Chitosan; Na-TPP: sodium tripolyphosphate; PDI: polydispersity index; SD: standard deviation.

**Table 2:** Optimization of midazolam (MDZ)-loaded CS NPs.

Formulation Code	Drug: Polymer Ratio	Conc. of CS (mg/mL)	Conc. of Na-TPP (mg/mL)	Mean Particle Size (nm) ± (SD)	Mean PDI ± (S.D)	EE (%)	DL (%)
F32 D1	1:1	1	1	241.20 ± 12.25	0.389 ± 0.056	88.68 ± 1.22	36.45 ± 2.14
F32 D2	2:1	1	1	320.10 ± 20.80	0.423 ± 0.028	77.67 ± 2.58	26.10 ± 1.84
F32 D3	3:1	1	1	380.96 ± 13.81	0.483 ± 0.014	68.46 ± 0.43	16.18 ± 2.01

EE: Entrapment efficiency; DL: drug loading.

**Table 3:** Pharmacokinetic parameters of MDZ formulation after intranasal (IN) and intravenous (IV) administration in rats.

Formulation	Organ/ Tissue	*C*_max_ (ng/mL)	*T*_max_ (h)	AUC 0–480 min (ng. min/mL)	AUC 0-Inf min (ng. min/mL)	*T*_1/2_ (min)	*K*_elim_ (min^−1^)
MDZ Sol IV	Brain	245.44 ± 12.83	1	1208.94	2559.19	592.8	0.070
	Blood	846.32 ± 22.01	0.5	2440.03	2950.26	172.2	0.241
MDZ Sol IN	Brain	211.67 ± 12.82	2	1036.78	2342.31	640.8	0.064
	Blood	209.83 ± 11.78	1	1094.46	3152.20	823.2	0.050
MDZ CSNPs IN	Brain	423.41 ± 10.23	2	1920.87	4673.52	782.4	0.053
	Blood	282.34 ± 31.45	2	1433.18	14792.37	4150.2	0.010

*C*_max_: Maximum plasma concentration; *T*_max_: time taken to reach *C*_max_; AUC_0-480_: area under the curve from 0 to 480 min); AUC_0-inf_: area under the curve from 0 to infinity); *T*_1/2_: elimination half-life; *K*_elim_: elimination rate constant.

**Table 4:** Distribution of MDZ solution (IV), MDZ solution (IN), and MDZ CS-NPs (IN) at different time intervals in Wister rats (*n* = 3).

Formulation	Organ/Tissue	0.5 h (ng/mL)	1 h (ng/mL)	2 h (ng/mL)	4 h (ng/mL)	8 h (ng/mL)
MDZ Solution (IV)	Brain	98.29 ± 16.44	245.44 ± 12.83	227.89 ± 11.76	156.33 ± 12.68	94.73 ± 13.81
	Blood	846.32 ± 22.01	626.65 ± 18.36	420.99 ± 11.31	293.44 ± 16.35	123.33 ± 14.22
MDZ Solution (IN)	Brain	87.99 ± 9.82	188.64 ± 11.69	211.67 ± 12.82	128.82 ± 18.94	84.67 ± 16.72
	Blood	106.82 ± 9.86	209.83 ± 11.78	182.32 ± 21.11	143.03 ± 19.44	103.91 ± 10.63
MDZ CS NPs (IN)	Brain	136.38 ± 10.71	293.27 ± 11.84	423.41 ± 10.23	246.36 ± 19.61	146.31 ± 14.85
	Blood	97.39 ± 11.65	187.33 ± 21.73	282.34 ± 31.45	192.36 ± 21.46	133.88 ± 19.26

**Table 5:** Brain/blood ratio for MDZ at different time intervals in Wister rats.

Formulation	Organ/Tissue	0.5 h	1 h	2 h	4 h	8 h
MDZ Soln IV	Brain/Blood	0.12	0.39	0.54	0.53	0.77
MDZ Soln IN	Brain/Blood	0.82	0.90	1.16	0.90	0.81
MDZ NPs IN	Brain/Blood	1.40	1.57	1.50	1.28	1.09

**Table 6:** Results of drug-targeting efficiency (DTE%) and direct transport percentage (DTP%) of MDZ formulation following IN administration.

Formulation	DTE%	DTP%
MDZ solution (IN)	191.373	49.13
MDZ-loaded CS NPs (IN)	270.707	63.09
